# Can Genetic Factors Compromise the Success of Dental Implants? A Systematic Review and Meta-Analysis

**DOI:** 10.3390/genes9090444

**Published:** 2018-09-06

**Authors:** Joel Ferreira Santiago Junior, Claudia Cristina Biguetti, Mariza Akemi Matsumoto, Guilherme Abu Halawa Kudo, Raquel Barroso Parra da Silva, Patrícia Pinto Saraiva, Walid D. Fakhouri

**Affiliations:** 1 Pró–Reitoria de Pesquisa e Pós-graduação (PRPPG), Universidade do Sagrado Coração, USC, 10–50 Irmã Arminda, Jardim Brasil, Bauru, SP 17011–160, Brazil; Guilherme_kudo@hotmail.com (G.A.H.K.); ppbau@uol.com.br (P.P.S.); 2 Department of Basic Sciences, São Paulo State University (Unesp), School of Dentistry, Araçatuba, SP 16015-050, Brazil; klaudiabiguetti@gmail.com (C.C.B.); vicmak.blv@terra.com.br (M.A.M.); raque_parra@hotmail.com (R.B.P.d.S.); 3 Center for Craniofacial Research, Department of Diagnostic and Biomedical Sciences, School of Dentistry, University of Texas Health Science Center at Houston, Houston, TX 77054, USA; 4 Department of Pediatrics, McGovern Medical School, University of Texas Health Science Center, Houston, TX 77030, USA

**Keywords:** genetic factors, dental implants, bone quality, bone regeneration, survival rate

## Abstract

Dental implants provide a predictable treatment option for partial and complete edentulism via the placement of a fixed permanent artificial root to support prosthetic dental crowns. Despite the high survival rates, long-term dental implant failures are still reported, leading to implant removals and additional financial and health burdens. While extrinsic factors that improve the success rate of implants have been well explored, the impact of genetic factors on this matter is poorly understood. A systematic review and meta-analysis study was conducted to determine whether genetic factors contribute to an increased risk of dental implant failures. A comprehensive search for peer-reviewed articles on dental implants and genetic factors was performed using various literature database libraries. The study design was conducted according to Preferred Reporting Items for Systematic Reviews and Meta-Analysis (PRISMA) guidelines, and the obtained records were registered in the International Prospective Register of Systematic Reviews (PROSPERO) database. According to the exclusion/inclusion criteria, 13 studies were eligible for this study out of 809 articles. The meta-analysis of the combined association studies of DNA variations and dental implants did not indicate an increased risk for implant failure due to DNA variations in *IL-1B*, *IL-10* and *TNF-α*. This study emphasizes the need for larger randomized controlled clinical trials to inform clinicians and patients about the role of genetic factors on dental implant survival and the success rate in healthy and compromised patients.

## 1. Introduction

Association studies between common DNA variations and human diseases have been proven very useful for identifying genetic factors that increase the risk or provide protection to human complex diseases [1,2]. DNA variations can modify gene expression and function which can increase susceptibility for a disease and affect a person’s phenotype. Each individual carries on average 3.55–4.6 million single nucleotide polymorphisms (SNPs) which can be defined as an alteration of a single nucleotide base that occurs in at least >0.5% of the human population [1,2,3,4,5]. Therefore, applying this approach should be considered to determine the contribution of genetic factors when it comes to dental implant failure and bone loss [1,3,4,6,7,8]. Previous studies reported that various systemic diseases can lead to implant failure, including the influence of immune system regulation on bone metabolism and bone density [7,9,10]. It has been emphasized that the host–implant interaction and the response to foreign objects could be a major cause for severe marginal loss and consequent implant failure rather than a secondary biofilm-mediated infection [10]. Furthermore, recent reports indicated that poor bone quality can reduce dental implant survival [11,12,13]. In this context, knowledge of the genetic factors that influence osseointegration and the possible longevity of dental implants is pertinent for investigation in the field of implantology, in order to identify intrinsic risk factors [3]. Notably, research involving the host response regarding the marginal bone loss in the osseointegrated interface remains unexplored.

Failure of osseointegration occurs due to multifactorial conditions, including individual susceptibility or risk factors. Failure can occur even under proper conditions of bone tissue due to possible host immune responses [7,14,15]. Considering this possibility, it is important to investigate the intrinsic characteristics of individuals who experience dental implant failures to identify genetic factors that influence osseointegration [4]. The immune system is important in regulating the balance of cytokines and chemokines during inflammatory conditions. Depending on the local condition, the presence of pathogens in the oral cavity can alter the components of the immune response such as the cytokines and growth factors involved in the regulation of the healing process [15,16]. In the case of dental implants, the surgical procedures stimulate an initial inflammatory response to the implanted artificial root by the production of several types of cytokines and other mediators, such as interleukins [17]. Different types of interleukins play an important role in bone remodeling by inducing bone resorption (e.g., IL-6), or stimulating bone formation (e.g., IL-10) [1,4,7,18]. While some cytokines act as an anti-inflammatory profile, such as IL-10, other molecules and cytokines such as IL-2 and IL-6 are involved with pro-inflammatory activity and bone loss [19].

Polymorphisms in the promoters of *IL-2* and *IL-6* genes were associated with an increase in the expression of both cytokines and with the development of chronic diseases such as periodontitis [1]. High levels of inflammatory mediators have been detected in patients with local infections, which would indicate that local infections could aggravate marginal bone loss and threaten dental implant success [20]. Moreover, inflammatory conditions can affect the balance of other molecules involved in bone matrix homeostasis, such as matrix metalloproteinases (MMPs) and their tissue inhibitors (TIMPs) [3]. Polymorphisms within MMP genes are significantly associated with a number of dental and bone pathologies, and their presence in the peri-implant fluid could trigger a peri-implant disease with further bone loss [3]. It is not fully established how the interplay of these factors occurs, therefore further studies in this research area are required to determine whether synergistic or antagonistic interactions among these molecules play a role in osseointegration or bone loss. While multiple studies have investigated the effect of genetic factors on dental implant survival rates [1,3,4,21], no large or randomized controlled clinical studies have been conducted to define the contribution of multiple DNA variations and genetic factors to dental implant failure. Therefore, it is important to understand the underlying molecular mechanism that leads to dental implant failure to improve the clinical outcome by preventing or developing targeted therapy. Thus, the aim of the present study is to analyze the relation between genetics and implant failures by means of a systematic review and meta-analysis. The null hypothesis is that genetic factors do not influence dental implant survival rate, while the alternative hypothesis is that certain genetic factors increase the risk for dental implant failure. 

## 2. Methodology

### 2.1. Standardized Criteria and Type of Study

This systematic review and meta-analysis study was designed according to the established criteria by Cochrane collaboration for the design of the systematic review and meta-analysis [22]. The authors followed the Preferred Reporting Items for Systematic Reviews and Meta-Analysis (PRISMA) criteria [23], as well as the recently published models of systematic review to ensure the standardization of the data inclusion/exclusion criteria and analysis [13,24,25,26,27,28]. 

### 2.2. Registry Protocol

The data of this study was registered by the International Prospective Register of Systematic Reviews (PROSPERO) database under the number CRD42018088458. This data is publicly available for download at https://www.crd.york.ac.uk/prospero/display_record.php?RecordID=88458. 

### 2.3. Eligibility Criteria

The analysis was designed based on the PICO index as follows; (1) Population: patients who received oral rehabilitation; (2) Intervention/Exposure: effects of genetic factors on dental implant failure; (3) Comparison: group that lost dental implants vs. group that did not lose installed implants within six months of treatment; (4) Outcomes: potential association between DNA variations and dental implant failure with other characteristic phenotypes.

### 2.4. Inclusion/Exclusion Criteria and Cohort Size

#### 2.4.1. Inclusion Criteria

A literature search until February of 2018 was performed to select studies that contained the following criteria: (1) published in English; (2) minimum clinical follow up of six months of retrospective and prospective studies, controlled and randomized clinical trials; (3) adult patients (≥18 years) that received dental implants were considered. 

#### 2.4.2. Exclusion Criteria

In vitro, animal studies, clinical reports, reviews, non-controlled or incomplete data were not considered and consequently excluded. Clinical studies with a greater focus on smoking, periodontal disease, or systemic diseases were not included in the systematic review. Clinical studies with less than ten patients were excluded.

### 2.5. Search Strategy

A search for articles published until February, 2018, was made of the PubMed, Cochrane, and Web of Science databases. Boolean operators based on MeSH and PubMed included the following: “Dental Implants” and “bone genes”, “dental implants” and “genetic risk factors”; “dental implants failure” and “genetic risk factors”. Related search by PubMed was: (“dental implants” [MeSH Terms] OR (“dental” [All Fields] AND “implants” [All Fields]) OR “dental implants” [All Fields]) AND (“bone and bones” [MeSH Terms] OR (“bone” [All Fields] AND “bones” [All Fields]) OR “bone and bones” [All Fields] OR “bone” [All Fields]) AND (“genes” [MeSH Terms] OR “genes” [All Fields]); (“genetic therapy” [MeSH Terms] OR (“genetic” [All Fields] AND “therapy” [All Fields]) OR “genetic therapy” [All Fields] OR “genetic” [All Fields]) AND (“risk factors” [MeSH Terms] OR (“risk” [All Fields] AND “factors” [All Fields]) OR “risk factors” [All Fields]) AND (“dental implants” [MeSH Terms] OR (“dental” [All Fields] AND “implants” [All Fields]) OR “dental implants” [All Fields]) AND failure [All Fields]; (“dental implants” [MeSH Terms] OR (“dental” [All Fields] AND “implants” [All Fields]) OR “dental implants” [All Fields]) AND failure [All Fields] AND (“genetic therapy” [MeSH Terms] OR (“genetic” [All Fields] AND “therapy” [All Fields]) OR “genetic therapy” [All Fields] OR “genetic” [All Fields]) AND (“risk factors” [MeSH Terms] OR (“risk” [All Fields] AND “factors” [All Fields]) OR “risk factors” [All Fields]).

A manual search from September 2017 until February 2018 was conducted for the following journals: *Implantology*, *Clinical Implant Dentistry and Related Research, Clinical Oral Implants Research, European Journal of Oral Implantology*, *Implant Dentistry, International Journal of Oral and Maxillofacial Implants*, *International Journal of Oral and Maxillofacial Surgery*, *International Journal of Periodontics and Restorative Dentistry*, *International Journal of Prosthodontics*, *Journal of Clinical Periodontology*, *Journal of Dental Research*, *Journal of Oral Implantology*, *Journal of Oral and Maxillofacial Surgery*, *Journal of Oral Rehabilitation*, *Journal of Periodontal Research*, *Journal of Periodontology*, and *Journal of Prosthetic Dentistry*. Additionally, a manual search was conducted in the references of included articles.

### 2.6. Data Collection Process

Three previously calibrated reviewers (R.B.P.S., M.A.M. and J.F.S.J.) selected the articles and performed the data collection. Discrepancies in the analysis were solved in a consensus meeting for analysis of the selected titles and abstracts, with an agreement test value for the selected articles in the three databases. In order to decrease bias in the selection of the articles, authors G.A.H.K. and C.B. participated in the selection of the sample, data collection and examination of the databases as well. Consensus meetings for the selection of each article within the selected sample pool were held on weekly bases (November, December 2017 and February 2018). The group and other researchers worked together to consolidate the analysis of the topics (P.P.S. and W.D.F.).

### 2.7. Items of Extracted Data

Data extracted from studies that passed the inclusion criteria were analyzed and the main standardized information were obtained as follows: (1) author; (2) year of publication; (3) country and origin of the study; (4) number of patients; (5) analyzed group of patients; (6) mean age of the patients; (7) number and sites of implants; (8) trade mark of the implants; (9) implants failure; (10) main data of the implants; (11) data of periodontal evaluation; (12) studied variables; (13) methodologies; (14) rate of peri-implant bone loss; (15) follow-up period of each study. Data were collected using a standardized file built in Excel software.

### 2.8. Evaluation of the Study Quality and Risk of Bias

The selected clinical studies were evaluated based on the consideration of their methodology approach on a bias scale by the National Health and Medical Research Council (NHMRC—Australian Government). The bias scale allows for evidence levels of different categorical studies (intervention, diagnostic accuracy, prognosis, etiology, screening intervention). Therefore, the NHMRC establishes a hierarchy of studies, classifying clinical studies at different levels. For intervention studies: I-systematic review; II-Randomized controlled study; III-1 pseudorandomised controlled study; III-2 clinical study with a group control; III-3 a comparative study without control group; IV case series [28,29,30]. 

### 2.9. Measurements and Statistical Analysis

The quantitative data collected from the articles was tabulated for the analysis of odds ratio (OR) with a correspondent 95% confidence interval (CI). For all analysis, significant values were considered as *p* < 0.05. Reviewer Manager 5.3 (Cochrane Group) was used for the meta-analysis and graphic elaboration. 

### 2.10. Anticipated Outcome

#### 2.10.1. Primary Outcome

The primary outcome was to analyze if there is a significant association between genetic factors and the failure of dental implants based on previously published studies that passed the inclusion criteria.

#### 2.10.2. Risk of Bias of Quantitative Data 

A fixed-effects model was applied in case no significant differences were observed in the data. Alternatively, a random effects model was applied in case significant differences (high heterogeneity among the tests) were observed. Heterogeneity was considered significant at *p* < 0.1 and was evaluated using the Q (x^2^) test and *I*^2^ value. Statistical *I*^2^ value was used in the analysis of heterogeneity variations, and values above 75 (0–100) were considered to indicate significant heterogeneity [24,31,32,33].

#### 2.10.3. Additional Analysis 

Sensitivity tests for the analysis of patient subgroups and the allele frequency of different types of alleles for the genes *ILs* and *TNFα* were made in order to avoid potential heterogeneity due to different groups of patients (failure vs. control group) [13,24]. 

## 3. Results

**Figure 1:**
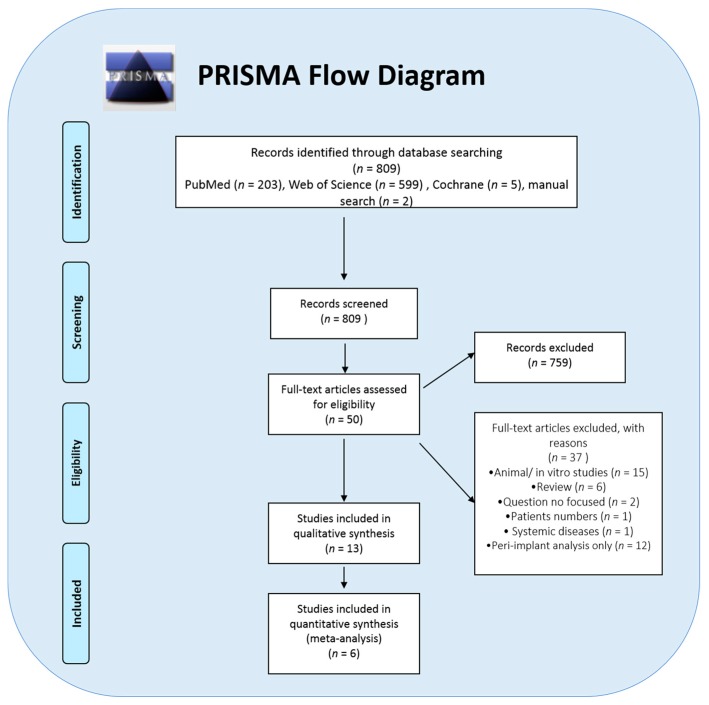
A flowchart representing the literature search, screening, eligibility and selection of this study.

A total of 809 articles were identified from the three online databases searches. After applying the inclusion/exclusion criteria, 50 articles were eligible for full-text assessment. After a complete assessment, 13 articles fulfilled the inclusion criteria for qualitative analysis and 6 studies for meta-analysis, as shown in Figure 1. 

Eleven out of the 13 selected studies were from South America (Brazil) [1,3,4,6,7,8,17,20,21,33,34], and two from Europe (Turkey and Portugal) [15,18]. Some studies reported the origin of the patients which can be used for further stratification of the data [4,8,33,34]. Out of the 13 analyzed articles, 12 were clinical and prospective studies [1,3,4,7,8,15,17,18,20,21,33,34], and they performed genomic DNA extraction and genetic testing on their patients and control group using saliva samples. In addition, these studies conducted a follow-up with patients that presented with failure or success of implants in a period that varied with a minimum of six months. One study was classified as retrospective [6]. 

For statistical analysis different kinds of cellular mediators were evaluated based on PICO criteria, with a focus on selecting studies that provided data on genetic factors that can lead to implant failure, such as vitamin D receptor polymorphism D [7], interleukins; *IL-1B* [4], *IL1A* and *IL1B* [18,35], *IL1B* and *IL1RN* [21], *IL-2* [1], *IL-4* [17] and *IL-10* [6,15,34], Tumor necrosis factor-α; *TNFα* [20], metalloproteinase-8; *MMP8* [3], growth factor β1; *GFβ1* [8], *RANKL* [6]. The selected studies presented similar related methodologies to process their samples, however, there were several distinct differences. Nine of the studies used epithelial cells extracted from the oral mucosa, amplified by polymerase chain reaction (PCR) and then analyzed by restriction fragment length polymorphism (RFLP) [1,3,4,7,8,17,20,21,34]. Two other studies used amplification refractory mutation system coupled with PCR [6,15]. One study used the variable number of tandem repeat (VNTR) [17], and another study used PCR for amplification followed by hybridization [18].

In relation to the impact of the genetic alterations on dental implants survival, only one study about the role of *MMP-8* presented significant effect for T allele in 76.25% of the study group (failed implants). The genotype T/T in 63.75% of the study group was indicative of early loss of the implants (*p* = 0.0011). The C/T genotype was found in 48% in the control group (with no implant failure), while in 63.75% of the patients was observed T/T genotype (*p* = 0.0009) [3]. Another study identified that in the *IL4* gene, *SNP-590*, the C allele was associated with implant loss (*p* = 0.0236, OR = 1.61, 95% CI: 1.1–2.4) for [17]. Finally, the study by Vaz et al. (2012) [18] analyzed *IL-1A* and *IL-1B* alterations and found that alleles 1 and 2 of both cytokines could be associated with success or failure of the dental implants. They also addressed environmental factors such as smoking and alcohol use and showed that there was no significant association with implant failures. The other studies did not indicate additional genetic risk factors for the failure of dental implants, as shown in Table 1.

### 3.1. Clinical Parameters

A total of 2130 patients combined from 13 studies were included for this systematic review and meta-analysis. Among them, only one study considered a single group of patients without including a control group [6]. The other studies used the comparison between two groups, one with successful implants (control group: 1291 patients), and the other with patients who presented with at least one implant failure (test or study group: 739 patients) [1,3,4,7,8,15,17,18,20,21,33,34]. In relation to the follow-up time, some studies limited the monitoring period of the control and study groups to one year [4,17,20]. One study examined the survival rate nine months post-operation [3], and another study chose a six months follow-up [8,34].

Although the minimum age considered for a patient was 18 years old, the mean age of participants was over 40 years in the eight selected studies [1,4,6,7,17,21,33,34]. Considering the number of implants installed, only three studies indicated the real quantity of those that achieved success or failure [7,17,35]. Ribeiro et al. (2017) [6] performed an objective analysis of 90 patients and a total of 245 implants, which impeded the identification of the exact number of failures. Alvim-Pereira et al., (2008) [7] emphasized that 50% of the failures occurred before 20 weeks (range: 0–237 weeks) from the implant date. Out of all the studies, only eight studies revealed the trademark of the dental implants [6,7,8,17,20,21,34,35]. Some studies highlighted clinical conditions which could reflect on the survival of dental implants. Alvim-Pereira et al. (2008) [7] reported the effects of the site of installation (maxillae and mandible, *p* = 0.003), posterior/anterior (*p* = 0.037), mean length of the implant (*p* = 0.001), primary stability (*p* = 0.001), surgical technique (*p* = 0.016), the quality of bone tissue (*p* = 0.049), and edentulism (*p* = 0.009) on dental implant survival. Dirschnabel et al. (2011) [4] also related the loss of implants as due to the edentulism (*p* = 0.019), site of implant installation (*p* = 0.001) and medical/systemic conditions of the patient (*p* = 0.04). Similarly, Pigossi et al. (2014) [17] identified edentulism (*p* = 0.031), maxillae/mandible (*p* = 0.003), anterior/posterior regions (*p* = 0.037), primary stability (*p* = 0.0010, and implant load (*p* = 0.001) as factors relating to implant survival. Montes et al. (2009) [21] highlighted that the genotype of *IL1RN* (intron 2) was associated with failure of implants in individuals with multiple dental implant losses in addition to factors such as edentulism and the number of teeth present. In addition, some studies analyzed the periodontal condition of the remaining teeth, detecting significant differences in probing depth index in comparison to the control groups, (*p* = 0.002 [16], *p* = 0.011 [6], *p* = 0.005 [4], *p* = 0.011 [20]). In the study of Pigossi et al. (2012) [34], the statistical analysis indicated that neither smoking nor other variables like gender, dental mobility, rheumatoid disease, cardiovascular disease, hypertension, medical treatment, and use of nonsteroidal anti-inflammatory drugs (NSAID) or steroidal anti-inflammatory drug, were associated with dental implant failure. Other clinical periodontal variables did not reveal any significant differences, as shown in Table 2.

### 3.2. Meta-Analysis Outcome

Studies that analyzed the same allele of the same gene of interest were grouped together to increase the power of statistical analysis and the level of significance by comparing the control groups with no dental implant failure (control group) and the group with implant failure (study group). An increase of power analysis can be achieved by having a larger sample size of experimental and control groups with similar environmental conditions. Therefore, this study sought to combine all related studies that analyzed the association between DNA variations of genetic factors and dental implant failure. For each DNA allele, two studies were combined for the frequency distribution of genetic alleles. A high heterogeneity in the frequencies of the alleles and OR across the individual studies (*I*^2^ > 75%, *p* < 0.1) was not observed in this study. Acceptable measures (*I*^2^) were identified for study group of *IL10* (Failure group), *IL1B* (Control G vs. Failure G), *IL10* (Control G vs. Failure G), *TNF-α* (Control G vs. Failure G).

**Figure 2:**
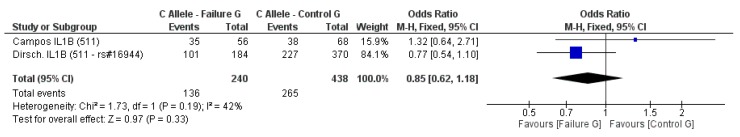
Odds ratio (OR) and forest plot for the prevalence of *IL1B-C511* allele in the failure group (at least one implant failure) vs. control group (without implant failure). The OR of the combined data crossed the line of no difference.

**Figure 3:**
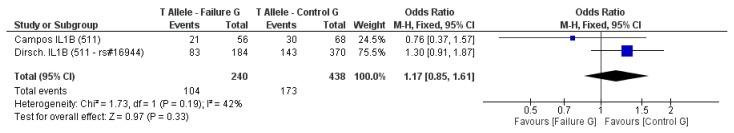
Odds ratio and forest plot for the prevalence of the *IL1B-T511* allele in the failure group (at least one implant failure) vs. control group (without implant failure). The OR of the combined data crossed the line of no difference.

The genetic data of two studies on the role of *IL1B* on dental implant failure were combined because they used the same SNP (*rs16944*) or allele (*IL1B-C511*) of *IL1B*. The genetic data was divided into four subgroups including the prevalence of *IL1B-C511* allele in study (failure) group and control group, and the prevalence of the *IL1B-T511* allele in the failure group and control group. The subgroups for each allele were combined from the two individual studies and analyzed for an association between study groups from both studies and dental implant failure [4,35]. Analysis of the two subgroups of *IL1B-C511* in the study group (failure G) and control group (control G) shows a pooled OR of 0.85, 95% CI of 0.62–1.18, and *p* value 0.33, indicating no significant association in carrying this allele and increase risk for implant failure (Figure 2). Similarly, no significant association between the *T511* allele and implant failure was identified compared to the control group with a pooled OR of 1.17 (95% CI: 0.85–1.61), *p* = 0.33 (Figure 3). The confidence intervals of both pooled ORs crossed the line of no effect indicating that both alleles do not increase the risk for dental implant failure under these circumstances of both studies.

**Figure 4:**
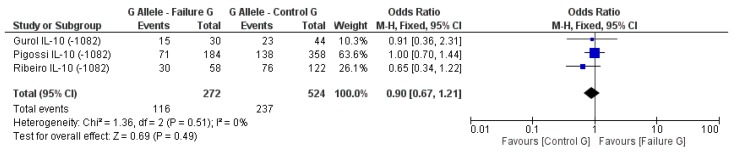
Odds ratio and forest plot for the prevalence of *IL10-G1082* allele in the failure group (at least one implant failure) vs. control group (without implant failure). The OR of the combined data crossed the line of no difference.

**Figure 5:**
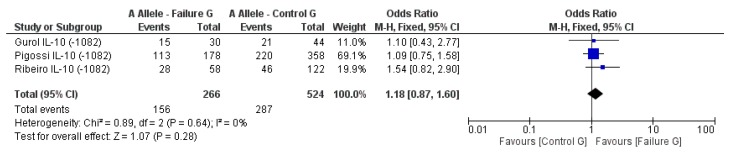
Odds ratio and forest plot for the prevalence of *IL10-A1082* allele in the failure group (at least one implant failure) vs. control group (without implant failure). The OR of the combined data crossed the line of no difference.

Another three studies attempted to determine whether DNA variations in *IL-10* are associated with dental failure compared to control group with no dental failures [6,15,34]. The statistical analysis of the association showed a pooled OR of 0.90 (95% CI: 0.67–1.21), *p* = 0.49, indicating no significant odds for the presence of the G allele in any of the groups (Figure 4). Furthermore, no significant difference in A allele expression was identified for both groups, the pooled OR was 1.18 (95% CI: 0.87–1.60), *p* = 0.28 (Figure 5).

**Figure 6:**
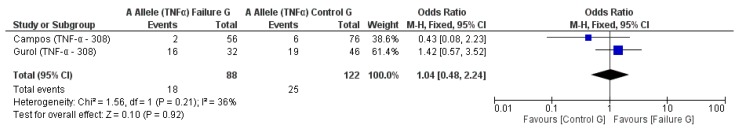
Odds ratio and forest plot for the prevalence of *TNF-α-A308* allele in the failure group (at least one implant failure) vs. control group (without implant failure). The OR of the combined data crossed the line of no difference.

**Figure 7:**
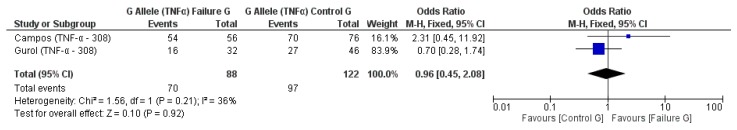
Odds ratio and forest plot for the prevalence of *TNF-α-G308* allele in the failure group (at least one implant failure) vs. control group (without implant failure). The OR of the combined data crossed the line of no difference.

Finally, two studies showed a comparison between the failure and control groups for *TNF-α* [14,19], in the analysis of the comparison between groups the pooled OR was 1.04 (95% CI: 0.48–2.24), *p* = 0.92, indicating no significant odds for the presence of the A allele in any of the groups (*TNF-α*: failure vs. control group), (Figure 6). As well as, no significant difference in G allele expression was identified for both groups, the pooled OR was 0.96 (95% CI: 0.45–2.08), *p* = 0.92 (Figure 7).

**Figure 8:**
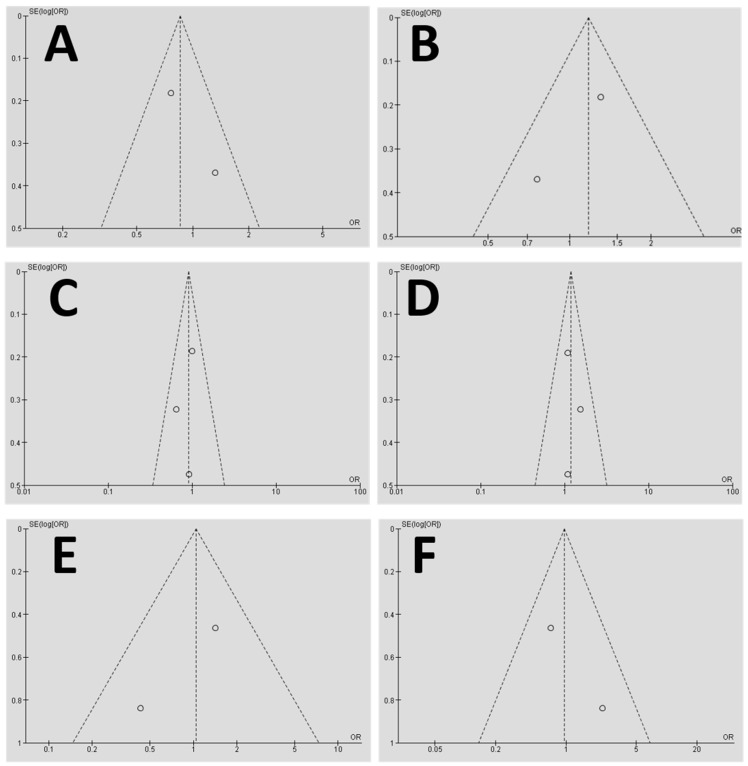
A funnel graph for the homogeneity representation of the meta-analysis. The data shows that the plotted homogeneity is acceptable for the DNA alleles used in the meta-analysis, including the *IL1B-C* allele study group (**A**), *IL1B-T* allele (**B**), *IL10-G* allele (**C**), *IL10-A* allele (**D**), *TNFα-A* allele (**E**), and *TNFα-G* allele (**F**). SE (log[OR]) = standard error of the natural logarithm of the odds ratio.

The homogeneity analysis for the comparison between study groups (failure) vs. the control group was performed among the selected publications in this study. The data shows low levels of heterogeneity for all the alleles included in the analysis (Figure 8A–F).

## 4. Discussion

Dental implants provide an excellent treatment option for patients with missing teeth via the replacement of tooth roots with a fixed permanent artificial root to match the natural ones and support prosthetic dental crowns. Despite the high success rate, implant failures are still common. In this context, long-term implant failure is generally a result of a severe bone marginal loss and bone resorption related to secondary infection [10]. Additionally, despite of some clinical similarities with tooth lost post periodontitis, recent clinical perspective studies in dentistry have shown that implant failure caused by severe bone resorption is not periodontitis-like disease and can be more related to the implant biomaterial characteristics and intrinsic patient factors [10]. The goal of this systematic review and meta-analysis is to delineate the effect of the genetic factors that can increase the risk for dental implant failure. This study was conducted following the established criteria for systematic reviews, registered in the PROSPERO base, and applied the PRISMA and PICO questions guidelines. While there are several factors that can influence the success of dental implants, like clinical and biomechanical factors, the impact of genetic risk factors has not been well investigated thus far [12,27,35,36]. In terms of relevant aspects for a satisfactory osseointegration of dental implants, previous study emphasized the importance of the inter-relation between primary stability and bone quality for the success of dental implants [7]. Furthermore, primary stability after implant installation allows an adequate bone–implant contact (BIC) for proper bone regeneration and integration. According to recent studies, material quality and surgical technique are also important factors for dental implant survival. Therefore, surgical techniques that can maximize BIC are important for enhancing the survival rate of dental implants, mainly in cases of low bone density [12,13,37]. Most of the implant failures occurred before a functional loading (around 81.3%), suggesting an important role of the host recipient site during the osseointegration process [17]. 

Due to the limitation of the included studies in this systematic review, it is important to note that none of these studies had indicated a sample randomization process, which might reflect a lower score on the bias scale of funnel graph analysis. Some of the selected studies presented initial sample randomization; however, a smaller number of the patients from those samples were chosen for their study and control groups [6,7,17,33]. Vaz et al. (2012) [18] performed sample calculations for the constituents of their test and control samples. In order to reduce possible biases in sample selection, including a large number of patients with randomized control trails that take into consideration a match for age, sex, ethnicity and gender among control and patients, some studies performed an equalization in order to homogenize the groups with a better division between smoking patients, ethnic groups, gender and age [4,7,17].

Bone peri-implant marginal loss can be aggravated by chronic or systemic diseases such as cardiovascular diseases, osteoporosis, diabetes, hepatitis, severe periodontal disease, chemo or radiotherapy, human immunodeficiency virus (HIV) positive infection, pregnancy or lactation, or even large bone reconstructions that compromise bone regeneration and integration. The included studies used in this systematic review selected patients who were healthy and with no systemic diseases [1,3,6,7,8,15,17,18,20]. However, one of the studies did not reveal the exclusion criteria of the patients [4]. Several clinical studies have focused on the role of interleukins in dental implant failure, because the clinical procedure of implant exhibits a higher level of interleukins after the first day of the implant installation due to normal local inflammation [1]. A higher cytokine activity in bone metabolism could enhance peri-implant bone loss leading to failure of the implant [1,38,39,40]. Unfortunately, this biological effect was not fully proven in this study because the meta-analysis of combined studies did not show an increase in the risk of dental implant failure in the presence of a specific type of allele in two *IL* genes. These results need to be analyzed with caution, since only two studies were considered in the sample: *IL1B* (C511T, rs16944) [35], *IL1B* (C511T) [4]. Therefore, additional well-designed studies should be performed in order to analyze the influence of *IL1B* on failure of dental implants.

A study by Campos et al. (2005) [1] analyzed DNA polymorphism in only one promoter of each *IL* gene, and reported that genes can present a number of polymorphic sites that act together. In this context, another publication suggests that genetic polymorphisms probably interfere with the process of osseointegration though a cumulative effect of multiple polymorphisms [3]. Another important point to consider is ethnicity because the majority of the selected studies are concentrated in Brazil, a country where its heterogeneity is composed mainly of Portuguese, Spanish and Italians [1,20]. The clinical study developed by Dirschanebel et al. (2011) [4] included 96.4% Caucasians individuals, likely from south-east Brazil, in their sample. It is possible that samples of other populations or ethnic groups can provide more insights and information [1]. 

Two clinical studies justified the lack of significant evidence due to sample size [18,20]. In fact, literature guidelines have stated that the clinical studies should be delineated, including a sample calculation [3,15], since it is difficult to compile a group of patients with implant loss and with specific exclusions such as smokers [3]. Furthermore, more studies should evaluate other cytokine genes that could influence an association between periodontal disease and the failure of dental implants [15]. One of the studies reported that the key for the success of osseointegration was not related to the level of cytokines production, but an advanced stage of bone formation could exist, as the calcification of organic matrix, which could influence bone remodeling [20]. The null hypothesis was partially accepted based on the meta-analysis and on the majority of the included studies since they did not identify significant associations of the analyzed genetic factors with the failure of dental implants [1,4,7,8,15,20]. However, a recent study reported that while only the +33C allele of the *IL-4* gene was associated with susceptibility of implant loss, when a SNP was included in the analysis of haplotype *IL-4*, a statistical difference was not identified [17]. It emphasizes the importance of a balanced analysis of the patients’ profiles in control and study groups, reinforcing the positive impact of randomized control studies. 

Multiple published studies highlighted the importance of considering the levels of marginal bone loss around dental implants for future clinical trials [4]. Furthermore, a recent systematic review study showed that there is a moderate association between peri-implantitis and higher expression of inflammatory cytokines, indicating that the evidence is still limited in this research area [41]. Finally, it is important that further controlled randomized studies be developed to establish a more precise answer to the question of this systematic review. The planning of the surgical technique, the study of anatomy, pre-existence risk factors, primary stability, quality and quantity of bone tissue had all been indicated as important factors involved in dental implant success. Regarding the minimum period of clinical follow-up with the patients, in this study it was considered to be six months, because this is considered a period of completion of the osseointegration. However, it is important to emphasize that clinical studies should consider a one-year clinical follow-up for the evaluation of long-term clinical conditions related to dental implant [13,24].

In conclusion, the meta-analysis of the combined genetic studies did not show increased risk or protection in dental implant failure due to DNA variations in *IL1B*, *IL10* and *TNFα* in study groups compared to control groups. Hence, there is no strong evidence that genetic factors can lead to the failure of oral rehabilitation with dental implants. It is important that additional randomized controlled studies with large sample size be conducted in order to conclusively determine whether there is a possible effect of genetic risk factors on implant failure and on marginal bone loss. These findings will help to identify those individuals with higher risk for dental implant loss facilitating the preparation of prevention strategies and individualized therapies in order to increase survival rates of oral rehabilitation with dental implants [3,4,6].

## Figures and Tables

**Table 1:** A summarized data of the articles selected in this study and the association between genetic factors and dental implant failure.

Selected Study	Type of Study	Study Place	Analyzed Variable	Results (Association of Genetic Factors on Implant Failure)
**Alvim-Pereira et al. 2008** [7]	Prospective	Brazil	Vitamin D Receptor (rs731236) *	n.s.
**Campos et al. 2005a** [1]	Prospective	Brazil	*IL-2* (T330G) *IL-6* (G174C) *	n.s.
**Campos et al. 2005b** [33]	Prospective	Brazil	*IL-1A* (−889) *IL-1B* (3953) *IL-1B* (−511C/T) *IL-RN* (intron 2) *	n.s.
**Campos et al. 2004** [20]	Prospective	Brazil	*TNF-α* (−308) *	n.s.
**Costa-Jr et al. 2013** [3]	Prospective, Multicentric	Brazil	*MMP-8* (C799T) *	Significant association of *MMP-8* with dental implant failure (*p* = 0.0011)
**Dirschnabel et al. 2011** [4]	Prospective	Brazil (S) **	*IL1B* (−511C/T) *	n.s.
**Dos Santos et al. 2004** [8]	Prospective	Brazil (SE & NE) **	Growth factor-β1 (C509T, G800A)	n.s.
**Gurol et al. 2011** [15]	Prospective	Turkey	*IL-10* (−1082A/G, 819, 592); *TNF-α* (308)	n.s. for *IL-10* and *TNF-α* alleles
**Montes et al. 2009** [21]	Prospective	Brazil	*IL-1B* (3954); *IL-1RN* (intron 2)	n.s. for genotype and allele frequencies of *IL1B* and *IL1RN* ^$^
**Pigossi et al. 2012** [34]	Prospective	Brazil	*IL-10* (−1082A, −819, −519)	n.s. for dental implants loss with genotypes (*p* > 0.05)
**Pigossi et al. 2014** [17]	Prospective	Brazil	*IL-4* (−590C/T; 33C/T)	Significant association of IL-4 C allele with implant loss (*p* = 0.0236, OR = 1.61, CI = 1.1–2.4).
**Ribeiro et al. 2017** [6]	Retrospective	Brazil	*IL-10* (−1082A/G) RANKL (−438A/G)	n.s. for *IL-10* and *RANKL* alleles
**Vaz et al. 2012** [18]	Prospective	Portugal	*IL1A* (−889) *IL1B* (3953)	Significant association of *IL-1A* and *IL-1B* alleles with dental implant failure

* = PCR–RFLP = polymerase chain reaction–restriction fragment length polymorphisms; ** = S (South), SE (Southeast), NE (Northeast); ^$^ = The number of teeth present was observed to influence implant loss, *p* = 0.027; CI = confidence interval. n.s. = not significant

**Table 2:** The size of the cohorts and the dental implants clinical data of the articles selected in this study.

Selected Studies	No. Patient	Groups	Ave. Age (years)	Implants	Trade Mark	Periodontal Evaluation (Partially Edentulous Patients)
**Alvim-Pereira et al. 2008** [7]	217	CG: 137 SG: 80	51.7 ± 11.3	CG:1232 SG: 135	Neodent^™^	Gingival Index: 0.64 ± 0.38 (CG), 0.65 ± 0.55 (SG). Plaque Index: 0.14 ± 0.26 (CG), 0.25 ± 0.42 (SG). Calculus Index: 0.08 ± 0.13 (CG), 0.14 ± 0.25 (SG). Probing attachment (mm): 2.68 ± 0.41 (CG), 2.52 ± 0.47 (SG). Clinical attachment (mm): 3.61 ± 0.76 (CG), 3.66 ± 1.10 (SG). Mobility (absence/presence): 98/13 (CG); 60/15 (SG)
**Campos et al. 2005a** [1]	74	CG: 40 SG: 34	43.8 49.3	NI	NI	NI
**Campos et al. 2005b** [33]	72	CG: 34 SG: 28	43.3 52.7	NI 97	3i^™^/ Conexão^™^	NI
**Campos et al. 2004** [20]	66	CG: 38 * SG: 28	NC	NI	3^™^/ Conexão^™^	NI
**Costa-Jr et al. 2013** [3]	180	CG:100 ** SG: 80	>18	NI	NI	NI
**Dirschnabel et al. 2011** [4]	277	CG:185 * SG: 92	53.6 ± 11.1	NI	NI	Gingival Index: 0.64 ± 0.37 (CG), 0.65 ± 0.53 (SG) Plaque Index: 0.12 ± 0.23 (CG), 0.23 ± 0.41 (SG) Calculus Index: 0.07 ± 0.12 (CG), 0.13 ± 0.24 (SG) Probing attachment (mm): 2.72 ± 0.46 (CG), 2.54 ± 0.47 (SG) Clinical attachment (mm): 3.62 ± 0.85 (CG), 3.66 ± 1.07 (SG) Mobility (absence/presence): 132/19 (CG), 70/15 (SG)
**Dos Santos et al. 2004** [8]	68	CG:40 ^$^ SG: 28	>18	NI	3i^™^/ Conexão^™^	NI
**Gurol et al. 2011** [15]	108	CG: 70 SG: 38	25–48	NI 16	NI	NI
**Montes et al. 2009** [21]	266	SG: 90 CG: 176	51.5 ± 11.5	1232 135	Neodent^™^	Gingival Index: 0.63 ± 0.38 (CG) and 0.65 ± 0.53 (SG) Plaque Index: 0.12 ± 0.24 (CG) and 0.24 ± 0.42 (SG) Calculus Index: 0.07 ± 0.12 (CG) and 0.13 ± 0.24 (SG). Probing attachment (mm): 2.72 ± 0.46 (CG) and 2.55 ± 0.47 (SG). Clinical attachment (mm): 3.61 ± 0.85 (CG) and 3.67 ± 1.07 (SG). Dental Mobility 18 (142) (CG); 16 (83) (SG)
**Pigossi et al. 2012** [34]	277	CG: 185 SG: 92	53.79 ± 11.3	NI	Neodent^™^	NI
**Pigossi et al. 2014** [17]	280	CG: 186 * SG: 94	56.1 ± 11.3	1232 135	Neodent^™^	Gingival Index: 0.63 ± 0.38 (CG) and 0.64 ± 0.28 (SG) Plaque Index: 0.12 ± 0.23 (CG) and 0.23 ± 0.41 (SG) Calculus Index: 0.07 ± 0.12 (CG) and 0.13 ± 0.24 (SG). Probing attachment level (mm): 2.72 ± 0.46 (CG) and 2.55 ± 0.47 (SG). Clinical attachment level (mm): 3.61 ± 0.85 (CG) and 3.67 ± 1.07 (SG). Dental Mobility: 19 (12.5) (CG); 16 (18.6) (SG)
**Ribeiro et al. 2017** [6]	90	1 Group	54.5	245	Straumann^™^	NI
**Vaz et al. 2012** [18]	155	CG: 100 SG: 55	NI	NI	NI	NI

NI = Not Informed; Neodent^™^ = Curitiba, PR, Brazil; 3i:3i^™^ = Palm Beach Gardens, Florida, USA; Straummann^™^ = Bern, Switzerland; SG = Study group; CG = Control group without loss of implant; SG = Study group with loss of at least one implant; * CG = patients without implant loss for one year of follow-up; ** CG = implants installed with at least nine months of follow-up; ^$^ CG = implants installed with at least 6 months of follow-up.
